# Community health care integration and fiscal expenditures: evidence from a synthetic control approach

**DOI:** 10.3389/fpubh.2025.1524984

**Published:** 2025-05-23

**Authors:** Yi Xiang, Guangjing Zhong, Xiaofeng Lei, Longqing Liu

**Affiliations:** ^1^College of Public Administration and Law, Hunan Agricultural University, Changsha, China; ^2^Institute of Agricultural Economics and Agricultural Zoning, Hunan Academy of Agricultural Sciences, Changsha, China; ^3^College of Economics and Management, Zhejiang Agriculture and Forestry University, Hangzhou, China

**Keywords:** community health care integration, government expenditure, health care expenditure, synthetic controls, robustness tests

## Abstract

**Background:**

Does the implementation of community healthcare integration policies affect fiscal expenditures as a key measure in addressing older healthcare demands and promoting healthy aging?

**Methods:**

This study utilizes the implementation of community healthcare integration in Yantai, a prefecture-level city in China, as a natural experiment to analyze its fiscal expenditure consequences by using the synthetic control approach.

**Results:**

Our empirical findings indicate that the program implementation markedly decreased fiscal expenditures in pilot cities. The decrease in expenditure is solely due to policy execution, with no confounding variables detected. The program implementation negatively impacted fiscal expenditures mainly by decreasing government healthcare spending in pilot zones.

**Conclusion:**

Consequently, in the ongoing effort to enhance community healthcare integration, local governments must devise context-tailored implementation strategies to attain sustainable growth, alleviate fiscal burdens, and improve older adult care services.

## 1 Introduction

The Nineteenth Central Committee’s Fifth Plenary Session proposed a national strategy to proactively address population aging, in which integrated community-home medical care policies constitute a pivotal mechanism for achieving active aging. This policy framework entails qualified medical institutions delivering coordinated healthcare services to support older adult individuals’ aging-in-community objectives. The community-based medical-nursing integration was chosen for analysis stems due to its nationwide scaling since 2015, with all 31 provincial-level administrative regions having initiated pilot programs—a policy diffusion pattern worthy of systematic examination. Phase-wise implementation commenced in 2016: 25 national pilot districts (e.g., Beijing’s Dongcheng) were designated in June, followed by 29 additional units (including Chaoyang District) in September, establishing a longitudinal policy experimentation framework. In the endeavor to construct a modernized socialist country, new mandates have emerged regarding the integration of medical care and nursing within the community. The 14th Five-Year Plan for Urban–Rural Community Service System Development mandates: (1) enhancing family-contracted service protocols, (2) augmenting medical personnel reserves, (3) optimizing service delivery mechanisms, and (4) institutionalizing medical-nursing integration. Then, the new wave of community healthcare integration will inevitably have an impact on government financial expenditure.

The early 20th century witnessed transformative reforms in health-social care integration, driven by international scholars whose ongoing academic engagement has yielded substantial contributions to this interdisciplinary domain. According to Wiles J L et al. (1), access to community-based elder care services allows older adults to get support from friends, family, neighbors, the government, and the community, all of which can significantly increase their level of satisfaction with the services they get. Williams A M et al. (2) categorized long-term care models into home care, institutional care, and hospice care. Lopreite and Zhu (3) utilized a Bayesian vector autoregressive (B-VAR) model to empirically examine the dynamic interrelations among China’s aging index, life expectancy, economic growth, and health expenditure from 1978 to 2016. The study revealed that economic growth stimulates increases in health expenditure while rising life expectancy drives up the aging index. A comparative analysis with the United States indicated that China is more profoundly impacted by population aging. To address these challenges, the authors proposed that China could establish and improve a three-tiered healthcare system, promote behavioral modifications, and execute reforms to the retirement system. Portrait F et al. (4) and Pulkki J et al. (5) found that gender, socio-economic status, cost of long-term care services, city size, and location affect the older adult’s selection of long-term care models. Lopreite M et al. (6) employed a bibliometric approach to conduct a longitudinal analysis of the correlation between population aging and social security expenditures from 2011 to 2021, the study revealed that demographic aging, compounded by chronic conditions such as diabetes and hypertension, has significantly intensified the urgency of older adults’ demand for community-based older adult care services. This research highlights that age-related health issues increase the necessity for localized, accessible support systems designed for aging populations by comprehensively mapping scholarly trends and empirical data using bibliometric methodologies. This finding underscores the critical role of community-level care infrastructure in addressing the dual pressures of an aging society and rising chronic disease burdens, advocating for policy alignments that prioritize preventive and supportive services within social security frameworks. Lopreite et al. (6) conducted a longitudinal study on the correlation between aging and social security expenditures from 2011 to 2021 using a bibliometric approach. The research revealed that population aging, along with chronic diseases such as diabetes and hypertension, has made the demand for community-based older adult care services more pressing among older adults.

Comparative analysis of scholarly discourse reveals substantive convergence between Chinese and international research on community-based healthcare integration, particularly in theoretical frameworks, empirical investigations, and intervention strategies.

The current literature on community healthcare integration is broadly categorized into three types: joint operation of healthcare and nursing, integration of healthcare and nursing, and home-based care (7). Older people’s demand for community healthcare integration services and model selection are influenced by prerequisite, enabling and demand factors (8–11). At present, community healthcare and nursing integration has yielded remarkable outcomes, with the services encompassing life care services, healthcare services, and spiritual recreation services, which have a significant impact on enhancing the general sense of well-being and health well-being of the older adult (12). Community healthcare integration realizes the coordinated amalgamation of older adult care resources and medical resources, which can both provide effective care for the older adult (13) and reduce the medical costs of the older adult (14), and ultimately optimize the level of healthcare utilization (15) to achieve the older adult’s quality of life by leaps and bounds. Nonetheless, inadequacies persist in high-level planning, information integration, intersectoral collaboration, talent development, and the provision of medical and rehabilitative healthcare services. In addressing the aforementioned challenges, academics have suggested enhancements to China’s community-based medical care and rehabilitation service model by focusing on the fortification of integrated management, the optimization of information technology utilization (9), the enhancement of team development (16), and the implementation of rigorous scientific oversight (17), respectively.

The above literature has laid a profound theoretical foundation for the subsequent research of this paper, yet the following deficiencies remain: first, the majority of the research on community health care integration takes micro-individuals as the analytical unit to examine the effects of the implementation of the community health care integration policy on the individual old persons, while neglecting the implications for local government. Second, the impact of community healthcare integration on financial expenditure is overlooked, resulting in an incomplete comprehension of community healthcare integration policies. Thirdly, the research methodology mostly adopts the double-difference (DID) method, but the DID method is susceptible to “selection bias” and fails to satisfy the assumption of randomness (18, 19).

The marginal contributions of this study are primarily twofold: First, from a macro perspective, it analyzes the fiscal impacts of implementing community-based integrated medical and older adult care policies on local governments, thereby enriching the literature on policy evaluation for such integrated care models. Second, employing the synthetic control method (SCM) proposed by Abadie and Gardeazabal (20), the research designates a policy-piloting city as the treatment group, assigns weights to non-policy cities based on the similarity of their data characteristics to construct a synthetic control group, and compares fiscal expenditure changes between the treatment and control groups from 2016 onward. The estimated policy effect is derived from the differential changes noted between these two groups. The synthetic control method has the following three advantages: First, the synthetic control method is a nonparametric estimation method, which is an extension of the traditional double-difference method (21). Second, the control group information is represented by the predictive variables of the experimental group prior to the implementation of the policy, thereby minimizing the subjective selection bias (22); at the same time, the aggregate weights of each city in the synthetic control group is equal to 1, which avoids excessive extrapolation of judgment (23). Third, the synthetic control method effectively demonstrates the policy consequences throughout different periods after policy intervention (24). Accordingly, this study takes the event of the implementation of the community health care integration policy in Yantai Prefecture-level city, Shandong Province, China, before and after 2015 as a quasi-natural experiment to assess its influence on government fiscal expenditure. The selection of Yantai Prefecture-level city as the unit of analysis is justified by two reasons: first, Yantai Prefecture-level city, as a pilot policy of community health care integration, applies the synthetic control method to analyze the impact of community health care integration on government fiscal expenditures. Second, among the 16 cities in Shandong Province, Yantai possesses the second highest proportion of older adult population aged 65 years and above at 18.12% (Source: Bulletin of the Seventh National Population Census of Shandong Province (No.4), Statistical Bureau of Shandong Province), surpassing Qingdao, which is also one of the first pilot cities for healthcare integration.

The other parts of the study are structured as follows: the second part is the policy context; The third part is the data source and estimation method; The fourth part is the analysis of empirical results, robustness test and theoretical analysis. The last part is the conclusions of the study and the recommendations for countermeasures.

## 2 Explanation of policy background

As a new model of senior care service, the combination of health care not only helps to deal with the health governance problems brought about by the aging phenomenon, but also helps to promote the supply-side structural reform of senior care services, which is an important direction for the construction of a healthy China (25). In 2013, the State Council promulgated the “Opinions of the State Council on Accelerating the Development of the Senior Care Service Industry, “which formally proposed the promotion of the integration of healthcare development, and the combination of health care was officially put on the government’s agenda. In 2016, the General Office of the National Health and Family Planning Commission released the Program for the Division of Key Tasks for the Integration of Medical and Nursing Care, offering explicit guidelines for the advancement of medical and nursing services. Subsequently, pilot initiatives for the unification of medical and nursing services at the national level have been implemented consecutively across various provinces. Yantai, a prefecture-level city, has been designated as one of the initial pilot sites for the integration of community health care in China. To systematically amalgamate health and pension resources, enhance the transformation and advancement of grassroots health care, and better address the health care and pension service needs of the older adult population, the city has issued the “Guiding Opinions from the Yantai Municipal People’s Government Office on Accelerating the Promotion of Health Care Integration.” This initiative aims to fully mobilize social capital, including manpower, culture, information, and materials, to achieve sustainable development in the integration of medical and nursing services. In March 2021, to expedite the establishment of a senior care service system that integrates home community institutions with medical care and recreational nursing services, the Yantai Municipal Commission of Health and Wellness developed and promulgated a “Division of Responsibilities for the Development of Medical and Nursing Care Integration,” tailored to the city’s context, which explicitly defines the responsibilities and obligations of governmental entities.

The extension of life expectancy of the population is a natural trend of social and economic development, scientific and technological progress and improvement of medical and health facilities, but the extension of life expectancy of the population does not mean the continuation of health (11). The combination of medical and nursing care is based on the background of population aging, the traditional older adult service resources and medical and health care resources of the separation of the older adult generally can only get basic life care services, the older adult health management, social interaction, psychological construction and other needs have not been fully paid attention to. The integration of medical and nursing care in older adult care represents an innovative model that simultaneously delivers both services to the older adult, significantly contributing to the realization of healthy aging and the establishment of a healthy China. Community medical and nursing combination has both the convenience and economy of family nursing and the professionalism and scale of institutional nursing (12). Older adult people prefer to receive care in familiar communities (26), and community healthcare integration is in line with Chinese cultural traditions of aging, in which older adult people living in the community or at home can enjoy professional care, nursing, and rehabilitation services while maintaining their original social relationships with their family members, friends, and neighbors (27). Since the implementation of the policy of combining community health care, the health care industry in Yantai Prefecture-level city has realized the coordinated development of branding, scaling, and chaining, and has established a four-in-one health care complex of “institution + community + home + medical care,” which is of great theoretical and practical significance.

## 3 Data sources and estimation methods

### 3.1 Data source

The study data are sourced all from the Shandong Statistical Yearbook spanning the years 2011 to 2021. Simultaneously, to guarantee the reliability and scientific validity of the estimation results during the sample selection process, it is essential to exclude cities that also implement the policy of integrating community medical care and older adult care (20), ultimately resulting in the selection of 14 sample cities. The implementation of synthetic control necessitates the identification of dependent and independent variables. This study utilized fiscal expenditure as a dependent variable to assess government fiscal outlays. It is important to note that while total fiscal expenditures in a Chinese region are distributed between central and local governments, the central government bears a comparatively lesser financial burden, with local governments assuming the majority of regional fiscal responsibilities. Consequently, the fiscal expenditures analyzed in this study pertain primarily to those incurred by municipal governments. The existing literature identifies gross domestic product (lngdp) (28), population (lnpopulation) (29), fiscal revenue (lnfrevenue) (30), household consumption level (lnconsumption) (31), medical and health expenditure (lnmedicalexp), and urban and rural community expenditure (lnurbancom) as predictors of government fiscal expenditure.

### 3.2 Estimation method

Since 2015, Yantai Prefecture-level city has embarked on a trial implementation of the policy initiative of combining community healthcare and nursing, and the changes in fiscal expenditures resulting from the implementation of this policy can be viewed as a natural experiment implemented in the Yantai region. The Prefecture-level city of Yantai in 2015 and subsequent periods can be divided into an experimental group, with other regions that have not implemented such a community healthcare integration policy serving as a control group, and the specific impact of the community healthcare integration policy on fiscal expenditures can be estimated by comparing the differences between the two groups; thus, this study employs longitudinal panel data.

A methodical analytical approach involves comparing the disparity in fiscal expenditures between Yantai Prefecture-level city and other regions following the implementation of the community health care integration policy, thereby elucidating the program’s impact on fiscal expenditures. The traditional double-difference method is based on this comparative logic, but several limitations have been pointed out in the text above. In fact, other cities across the country and Yantai differ significantly in the various factors that determine fiscal expenditures, and would differ from each other in terms of fiscal expenditures even without the implementation of the community health care integration policy.

Therefore, this study adopts the synthetic control method proposed by Abadie and Gardeazabal (20) to construct a reasonable control object that accurately aligns with the characteristics of the experimental group as a means of overcoming the problem of inherent differences between the experimental and control groups. Synthetic control methods have gained prominence in policy effectiveness evaluation both domestically and internationally, owing to their distinctive advantages. Abadie et al. (20) were the pioneers in employing this methodology, demonstrating that terrorism adversely affected GDP per capita in the Basque Country. Subsequently, Abadie et al. (32) utilized synthetic control methods to investigate the effects of a comprehensive tobacco control program on tobacco consumption in California; Kleven et al. (33) used the synthetic control method to explore the relationship between the top income tax rate and international migration of soccer players. Domestic scholars have also conducted a large number of studies using the synthetic control method. Wang Xianbin et al. (34) and Liu Naiquan et al. (35) showed that the administrative division adjustment and the expansion of the Yangtze River Delta significantly contribute to economic growth by using the synthetic control method.

In the analysis of the fiscal expenditure data of *J + 1* cities, it is concerned that the first Prefecture-level city (Yantai) implemented the policy of combining community medical care in the *T_0_* period. The remaining cities that did not implement this policy were used as a control group in order to compare the changes in the growth of fiscal expenditure in these cities during the *T* period. For each city *i* (Value range: *1, 2,…, J + 1*) and different time periods *t* (the value range is *1, 2,…, T*), $G_{\mathrm{it}}^{N}$ represents the financial expenditure of city *i* when the policy of combining community medical and nursing care is not implemented at time *t*, $G_{\mathrm{it}}^{I}$ represents the financial expenditure of city *i* after the policy is implemented at time *t*. Therefore, through $\alpha_{\mathrm{it}}=G_{\mathrm{it}}^{I}-G_{\mathrm{it}}^{N}$ , we can get the specific impact of the implementation of the community health care policy on financial expenditure. The synthetic control method assumes that when *t ≤ T_0_*, no matter whether the policy is implemented or not, the fiscal expenditure of all cities satisfies the equation $G_{\mathrm{it}}^{I}=G_{\mathrm{it}}^{N}$ ; When *T_0_ < t ≤ T*, the financial expenditure of the sample market meets $G_{\mathrm{it}}^{I}=\alpha_{\mathrm{it}}+G_{\mathrm{it}}^{N}$ . At the same time, the hypothesis of this study $P_{\mathrm{it}}=1$ indicates that the sample city has implemented the community health care combination policy, and $P_{\mathrm{it}}=0$ indicates that the community health care combination policy has not been implemented. Thus, the financial expenditure observed by city *i* at time *t* can be expressed as Equation 1:


(1) $$G_{\mathrm{it}}=G_{\mathrm{it}}^{N}+P_{\mathrm{it}}\alpha_{\mathrm{it}}$$

When *t ≤ T_0_*, the sample market $P_{\mathrm{it}}=0$ without the implementation of the community health care combination policy can obtain $G_{\mathrm{it}}=G_{\mathrm{it}}^{N}$ ; When *T_0_ < t ≤ T*, $\alpha_{\mathrm{it}}=G_{\mathrm{it}}^{I}-G_{\mathrm{it}}^{N}=G_{\mathrm{it}}-G_{\mathrm{it}}^{N}$ , where $G_{\mathrm{it}}$ can be observed, indicating the government financial expenditure after the implementation of the community medical care combination policy, $G_{\mathrm{it}}^{N}$ cannot be directly observed. To get to $\alpha_{\mathrm{it}}$ , you need to set up a “counterfactual” spending model:


(2) $$G_{\mathrm{it}}^{N}=\delta_{t}+\theta_{t}Z_{i}+\lambda_{t}\mu_{i}+\varepsilon_{\mathrm{it}}$$

Equation 2 represents the determining model of potential fiscal expenditure, where *δ_t_* represents the time fixed effect, *Z_i_* is the set of predictor variables not affected by the community health care combination policy, *λ_t_* is a common influence factor that cannot be directly observed in dimension *1 × F*, and *μ_i_* represents the regional fixed effect that cannot be directly observed in dimension *1 × F*. *ε_it_* is a random disturbance term for each sample city, and its expected value is zero. For Yantai, a Prefecture-level city that has not implemented the policy of combining community medical care and nursing care, its financial expenditure can be obtained by weighted comprehensive simulation of the control city. To do this, we need to determine a (*J + 1*) -dimensional non-negative weight vector W^*^ = (w^*^_2_, w^*^_3_ … w^*^_J + 1_), and the ownership weight is required to be positive, and the sum is equal to 1. According to this weight setting, the resultant control results obtained can be expressed as Equation 3:


(3) $$\sum_{j=2}^{J+1}w_{j}G_{\mathrm{jt}}=\delta_{t}+\theta_{t}\sum_{j=2}^{J+1}w_{j}Z_{j}+\lambda_{t}\sum_{j=2}^{J+1}w_{j}\upsilon_{j}+\sum_{j=2}^{J+1}w_{j}\varepsilon_{\mathrm{it}}$$

Suppose there is a set of vectors W^*^ = (w^*^_2_, w^*^_3_ … w^*^_J + 1_) satisfies Equations 4 and 5:


(4) $$\sum_{j=2}^{J+1}w_{j}^{*}G_{\mathrm{jt}}=G_{11},\ldots ,\sum_{i=2}^{J+1}w_{j}^{*}G_{jT_{0}}=G_{1T_{0}}$$

and


(5) $$\sum_{j=2}^{J+1}w_{j}^{*}Z_{j}=Z_{1}$$

If $\sum_{i=2}^{T_{0}}\lambda_{t}^{'}\lambda_{t}$ is non-singular, Equation 6 can be obtained:


(6) $$\begin{array}{l} G_{\mathrm{it}}^{N}-\sum_{j=2}^{J+1}w_{j}^{*}G_{\mathrm{jt}}=\sum_{j=2}^{J+1}w_{j}^{*}\sum_{S=1}^{T_{0}}\lambda_{t}{\left(\sum_{n=1}^{T_{0}}\lambda_{n}^{'}\lambda_{n}\right)}^{-1}\\ \lambda_{s}^{'}(\varepsilon_{\mathrm{js}}-\varepsilon_{1s})-\sum_{j=2}^{J+1}w_{j}^{*}(\varepsilon_{\mathrm{jt}}-\varepsilon_{1t}) \end{array}$$

Abadie et al. (32) pointed out that the equation tends to zero when *t > T_0_*. In the time period *T_0_ < t ≤ T*, $\sum_{j=2}^{J+1}w_{j}^{*}G_{\mathrm{jt}}$ can be regarded as an unbiased estimate of $G_{\mathrm{it}}^{N}$ to represent $G_{\mathrm{it}}^{N}$ , and $\alpha_{1t}=G_{\mathrm{it}}^{I}-G_{\mathrm{it}}^{N}=G_{\mathrm{it}}-\sum_{j=2}^{J+1}w_{j}^{*}G_{\mathrm{jt}}$ can be further derived to estimate $\alpha_{1t}$ . In order to ensure the validity of Equation 6, two preconditions must be met: on the one hand, the feature vector of the first region must be located in the convex combination of the feature vector sets of other regions; On the other hand, the weight vector $W^{*}$ is determined by minimizing the distance between $X_{1}$ and the weight vector XW0 , where $X_{1}$ represents the (*k × 1*) dimensional feature vector of the pilot city before the implementation of the community health care combination policy, and $X_{0}$ represents a (*k × j*) matrix, whose column *j* corresponds to the feature vector of region *j* before the implementation of the policy. The eigenvector here refers to any linear combination of factors affecting fiscal expenditure or variables of fiscal expenditure in the Equation 4. The distance function| | $X_{1}-X_{0}\mathrm{Wv}=\sqrt{{\left(X_{1}-X_{0}W\right)}^{'}V(X_{1}-X_{0}W)}$ , where *V* is a symmetric semi-definite matrix of (*k × k*), the selection of which will affect the estimation error. Referring to the research method of Abadie et al. (20) this study selects a symmetric semi-definite matrix *V* that can minimize the estimation error, aiming to make the growth path of the synthesized result as close as possible to the actual growth trajectory of pilot cities before the implementation of the community health care integration policy.

## 4 Empirical process and results

### 4.1 Evaluation result

Based on the estimation of the synthetic control method, the city of Yantai in China was selected as the experimental group, and the remaining 13 sample cities were integrated to form the synthetic Yantai as the control group. Table 1 lists the weight combinations that constitute the synthetic Yantai. According to the construction logic of the synthetic control method, Jinan accounts for 92.3% of the weights in the virtual Yantai control group, which is mainly due to the fact that Jinan is highly matched with Yantai in terms of core predictor variables. In terms of regional economic characteristics, as the capital city of Shandong Province, Jinan and Yantai are highly comparable in terms of key indicators such as total GDP (both cities rank among the top three cities in the province in terms of economic output) and population size (both cities have a resident population of more than 7 million). At the same time, both cities are core nodes of the Shandong Peninsula City Cluster, showing convergence in non-economic variables such as the intensity of policy radiation and the level of infrastructure. This multi-dimensional feature overlap enables the algorithm to automatically assign higher weights to Jinan, ensuring that the “virtual Yantai” before policy intervention is highly consistent with the economic trajectory of the real Yantai. Linyi’s 7.7% auxiliary weight may correspond to Yantai’s unique maritime economic attributes.

**Table 1:** Synthetic weight combination of Yantai.

City	Weight
Jinan	0.923
Linyi	0.077

Table 2 illustrates the contrast between the actual Yantai and the artificially generated Yantai, created using the synthetic control approach, concerning financial expenditure factors prior to the enactment of the community health care integration strategy in Yantai Prefecture-level city. The data comparison reveals a significant similarity between the authentic Yantai and the synthetic Yantai across multiple variables. The disparity between the two is minimal, regardless of whether considering real GDP, population size, total fiscal revenue, resident consumption levels, or numerous variables such as medical and healthcare expenditures and urban and rural community expenditures.

**Table 2:** Fitting and comparison of predictor variables.

Variable	Real Yantai	Synthetic Yantai
*lngdp (billions)*	8.557	8.436
*lnpopulation (ten thousand people)*	6.549	6.572
*lnfrevenue (ten thousand dollars)*	15.078	15.105
*lnconsumption (dollars)*	9.732	9.939
*lnmedicialexp (ten thousand dollars)*	12.725	12.725
*lnurbancom (ten thousand dollars)*	13.032	13.308

**Figure 1:**
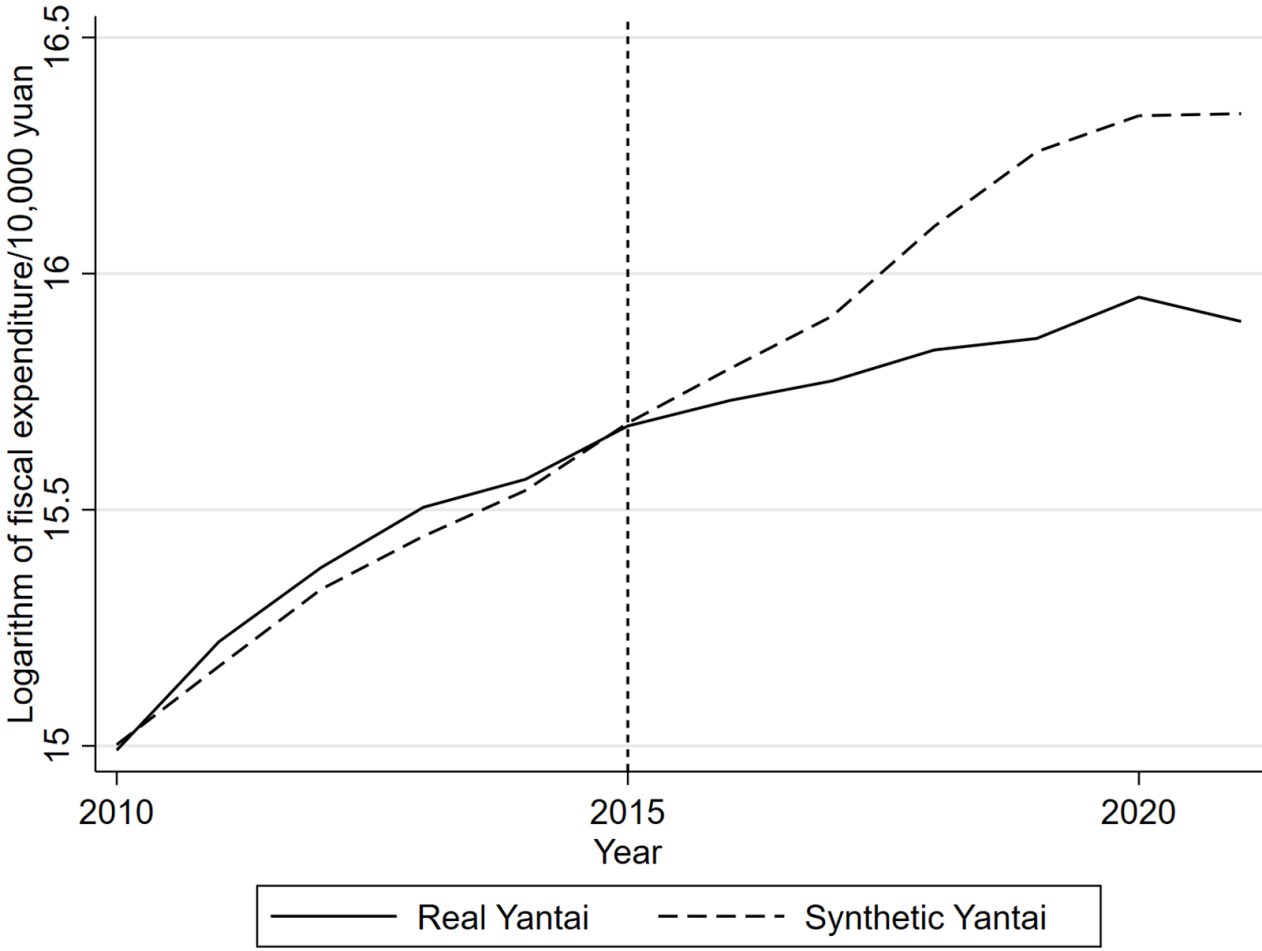
Real and synthetic financial expenditure pairs of Yantai.

In conclusion, the statistics shown in Table 2 indicate that the synthetic Yantai effectively replicates the actual conditions of Yantai prior to the enactment of the community health care integration policy, thereby rendering it appropriate for analyzing the specific impact of this policy on fiscal expenditures. Figure 1 clearly illustrates the trend of the logarithmic variation in actual fiscal expenditures of the real Yantai compared to the synthetic Yantai derived from the synthetic control approach prior to the policy’s adoption. The figure illustrates that the trajectories of fiscal expenditures prior to the implementation of the community health care integration policy are nearly indistinguishable, demonstrating that the synthetic Yantai effectively mirrors the growth pattern of the actual Yantai during the pre-implementation phase, thereby satisfying the fundamental criteria of the synthetic control method.

**Figure 2:**
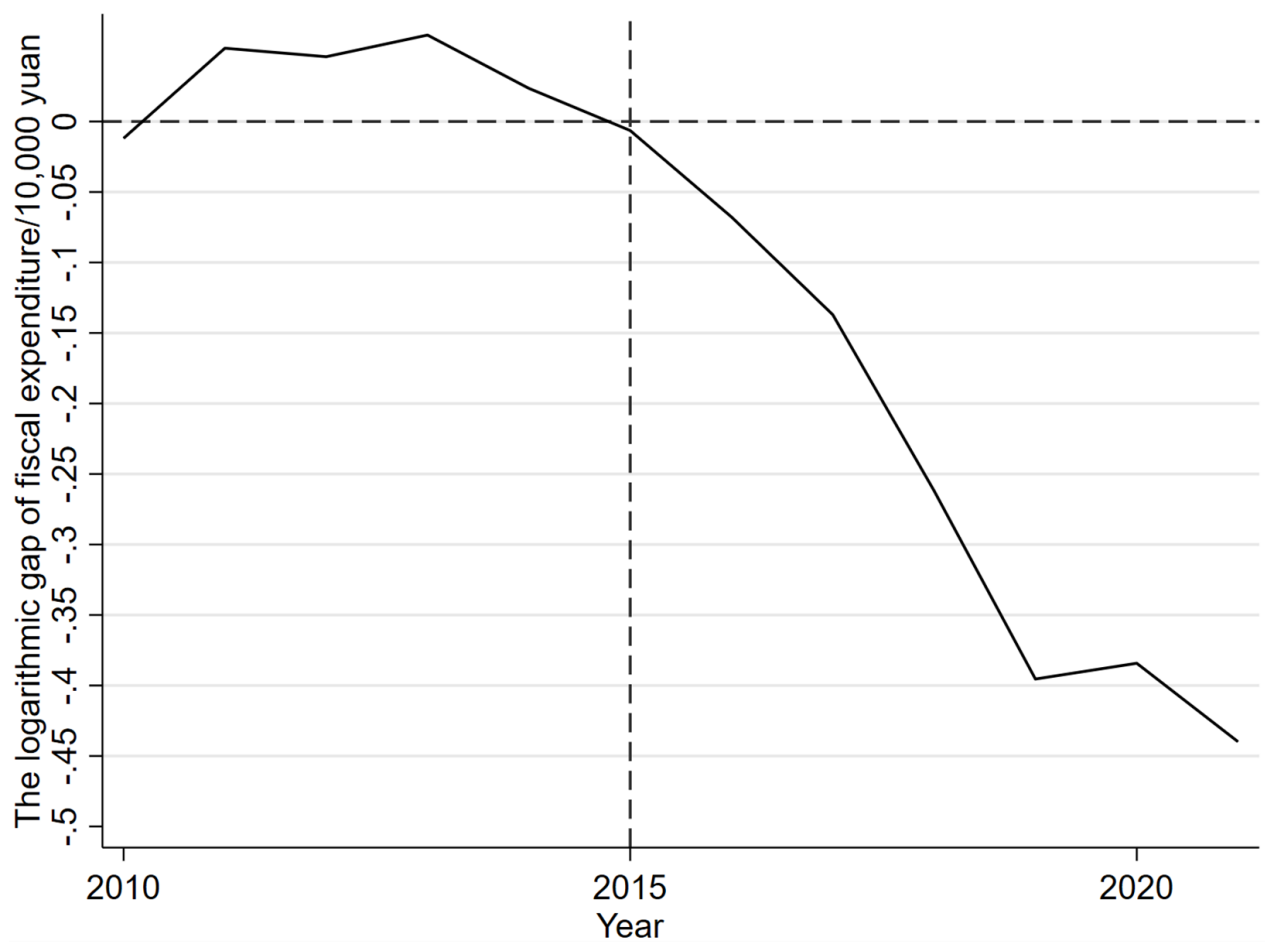
Logarithmic gap of financial expenditure between real and synthetic Yantai.

After 2015, the logarithm of real Yantai’s real fiscal expenditure began to be lower than the logarithm of synthetic Yantai’s real fiscal expenditure, and the difference between the two gradually increased, suggesting that the implementation of the community health care combination policy reduced the government’s fiscal expenditure. In order to more intuitively observe the impact of the implementation of the community health care integration policy on Yantai’s fiscal expenditures, this study calculates the fiscal expenditures of the real Yantai and the synthetic Yantai before and after the implementation of the community health care integration policy, and derives the difference between the two. As shown in Figure 2, the difference between the two logarithmic values fluctuates within the range of plus or minus 0.05 during the period from 2010 to 2015, after which the gap between the two breaks through the original range, and the gap continues to be negative with an expanding trend. It can be seen that after the implementation of the policy of combining community health care in Yantai City, the financial expenditures show a downward trend, indicating that the implementation of the policy of combining community health care helps to reduce financial expenditures.

### 4.2 Robustness test

After the synthetic control analysis, although it was found that there was a significant difference between the actual and synthetic Yantai financial expenditures, and that the financial expenditures showed a significant downward trend after the implementation of the community health care integration policy, did this difference originate from the implementation of the community health care integration policy? But is this difference due to the implementation of the community health care integration policy? Or is it just a chance phenomenon? That is, the results may be influenced by other unobserved factors, and the control group constructed by the synthetic control may not be able to fully replicate the potential evolutionary path of the experimental group. Therefore, uncertainty still exists in the results of the parameter estimates obtained from the synthetic controls. To ensure the reliability of the study, this study will validate the synthetic control estimates using three methods: double difference modeling, time placebo test and area placebo test to exclude the interference of chance and other factors.

#### 4.2.1 Double difference model

The double difference model is suitable for assessing the effectiveness and impact of policies after implementation and is able to remove variable selectivity bias that does not change over time (36). The double difference model constructed in this study is as follows:


(7) $$\text{Lnfependitur}e_{\mathrm{it}}=\alpha +\beta P_{\mathrm{it}}+\sum_{j=1}^{n}\mathrm{Ln}X_{\mathrm{ijt}}+\upsilon_{i}+\theta_{t}+\varepsilon_{\mathrm{ijt}}$$

In Equation 7, *i* is used to identify different cities, and *t* represents the corresponding year. The core variable *fependitureit* represents the fiscal expenditure of the *i* city in year *t* (ten thousand yuan). *X_ijt_* refers to the *jth* control variable that has an impact on the fiscal expenditure of the *i* city in a specific year *t*, including many factors such as the gross urban product, population size, fiscal revenue and residents’ consumption level. *P_it_* is a binary categorical variable with a value of *0* or *1*. If *P_it_* = 1, it means that in the year *t*, the *i* city has implemented the community health care combination policy; If *P_it_* = 0, it means that in the same year, the city has not implemented the community health care policy. *α* is a constant term to reflect the overall base effect of all cities; υ_i_ stands for regional fixed effect, which reflects the inherent differences between different cities. *θ_t_* represents a time-fixed effect, depicting the common trend effect on all urban fiscal expenditures over time. The $\varepsilon_{\mathrm{ijt}}$ represents the random error term, which covers other factors of random variation that are not adequately explained by the model.

**Figure 3:**
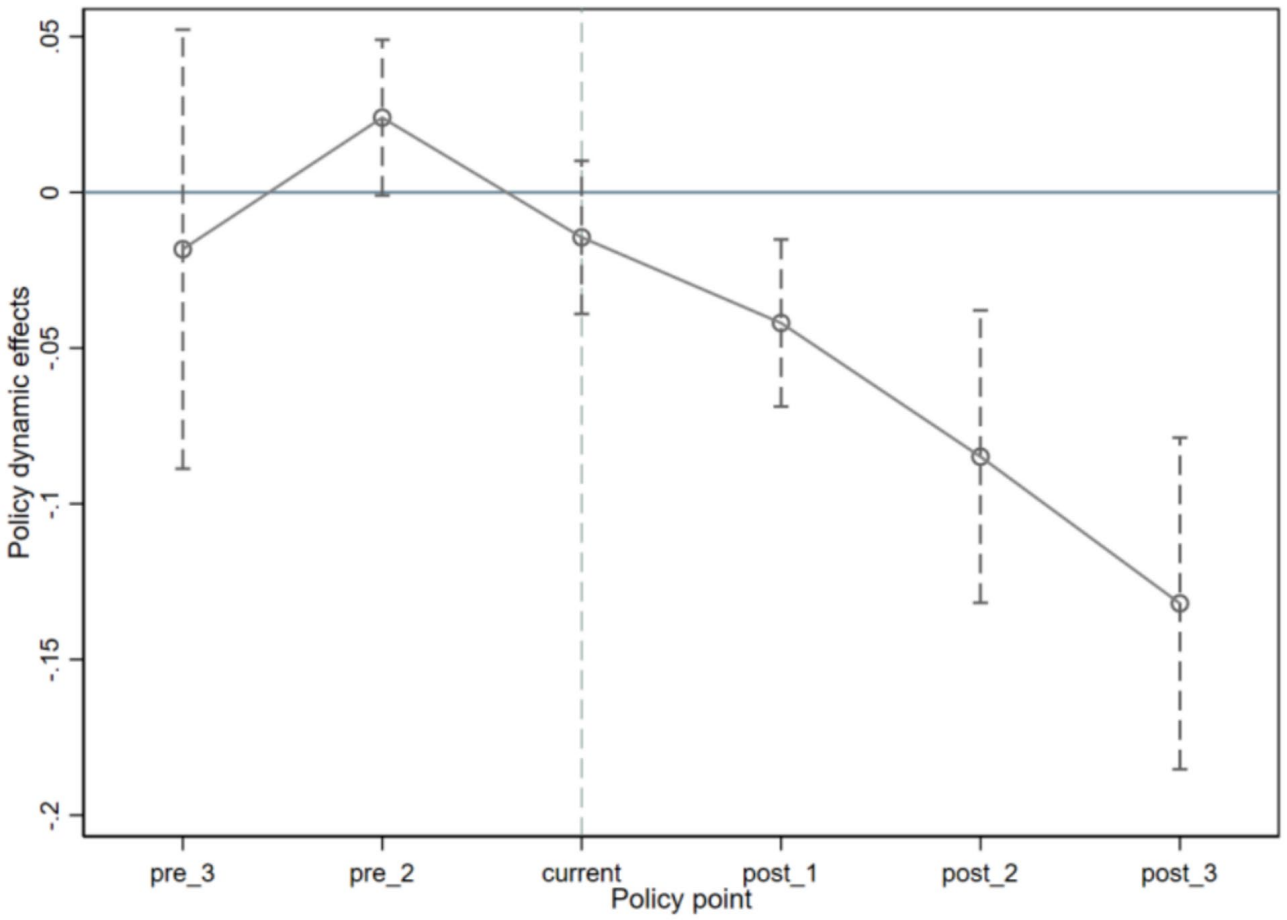
Parallel trend test results.

Table 3 demonstrates the effect analysis of the impact of the key variable of community health care integration on fiscal expenditures. From Model 1-Model 4, the effects of the community health care integration policy on fiscal expenditures are examined under the inclusion of time and area fixed effects, respectively. In Models 1 and 3 without the introduction of control variables, the effects of the community health care integration policy are validated at the 5% significance level whether it is implemented in the current period or in the lagged period, and the estimated coefficients are negative, implying that the policy significantly reduces fiscal expenditures in both the current and lagged periods. Further, in Model 2 and Model 4 with the addition of control variables, the specific effects of the current period implementation of the community health care integration policy and its lagged period implementation on fiscal expenditures are investigated respectively, and it is found that these two variables pass the test at 1% significance level, with the regression coefficient of the lagged period variable of −0.066, indicating that the effect of the community health care integration policy on reducing fiscal expenditures after 1 year of implementation is 6.6%. The results of Model 3 and Model 4 together reveal that the impact of this policy on fiscal expenditure is characterized by a lagged effect. This study hypothesizes that this lagged effect is mainly due to two reasons: first, it takes time for the actual effectiveness of the policy of combining community health care and nursing care to appear; second, there is also a certain time lag in the response of fiscal expenditure to policy adjustments. This point coincides with the trend shown in Figure 1, that is, the gap between the real Yantai Prefecture-level city fiscal expenditure and the fiscal expenditure after the synthetic treatment has shown a gradually expanding trend since 2015. The estimation by the double-difference method confirms that the implementation of the policy of combining community health care can indeed effectively cut fiscal expenditures, corroborating the reliability of the results obtained by the synthetic control method. In addition, this study also conducted a parallelism assumption test to observe whether there is a significant difference in fiscal expenditure between the two samples before and after the implementation of the policy by adding the interaction term of *treat* and year dummy variables in the model. The results show that the coefficients of *treat_it_ × time_it_* are both negative and the interaction term of *treat* with the ex ante year dummy variable is not significant, indicating that there is no significant difference between the treatment and control groups before the policy time point, while the interaction term of *treat* with the ex post year dummy variable is significantly negative, which fulfills the DID’s parallelism assumption as shown in Figure 3.

**Table 3:** Effect of community medical care combination on fiscal expenditure.

Variable	Dependent variable: logarithm of fiscal expenditure			
	1	2	3	4
*P_t_*	−0.087^**^ (0.034)	−0.101^***^ (0.020)	—	—
*P_t-1_*	—	—	−0.069^**^ (0.031)	−0.066^***^ (0.022)
*Control*	No	Yes	No	Yes
*Constant term*	14.457^***^ (0.021)	5.689^***^ (1.447)	14.457^***^ (0.019)	5.148^***^ (1.567)
*Regional fixed effect*	Yes	Yes	Yes	Yes
*Time-fixed effect*	Yes	Yes	Yes	Yes
*R^2^*	0.397	0.908	0.391	0.985
*Obs*	168	168	154	154

Figures outside parentheses are estimated coefficients, while figures inside parentheses are robust standard errors; *p < 0.1, ** *p* < 0.05, *** *p* < 0.01.

#### 4.2.2 Temporal placebo test

**Figure 4:**
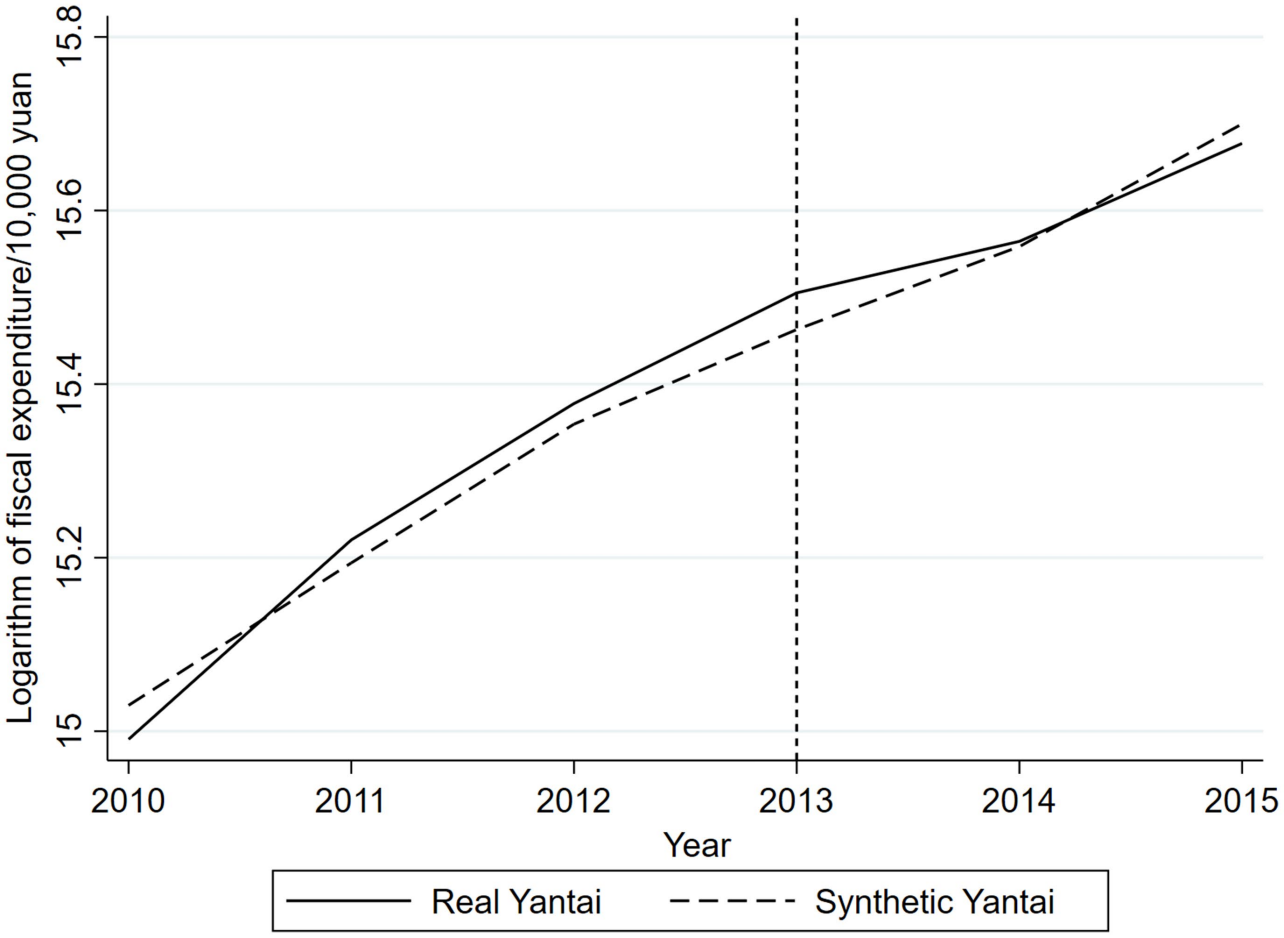
Results of temporal placebo test.

The core concept of placebo testing has similarities with simulation experiments. Drawing on the methodology of Abadie et al. (37) this study explores a time-series-based placebo test approach by adjusting the actual start time of the community health care integration policy in Yantai to a hypothetical earlier time period, and applying synthetic control methods again to analyze the effect of the policy on fiscal expenditures. In this process, we set the time of implementation of the policy in Yantai to be 2013 (as an intermediate time point before the implementation of the policy). Figure 4 illustrates the estimation results obtained using the synthetic control method.

According to the results of the time placebo test, the fiscal expenditure of the “virtual Yantai” constructed from the synthetic data is very close to that of the real Yantai before the implementation of the actual policy. This means that the hypothetical implementation of the community health care integration policy in 2013 did not lead to significant changes in fiscal expenditures, which verifies that the trend of fiscal expenditures reflected by the synthetic data can well match the trend of the real Yantai. The results shown in Figure 1 strongly reveal the specific impact of the community health care integration policy on fiscal expenditures, indicating that the synthetic control method has a certain degree of predictive efficacy.

#### 4.2.3 Regional placebo test

This study draws on the research method of Abadie et al. (32) to investigate the area placebo test. The core principle lies in the following: a city that has not actually implemented a community healthcare integration policy is selected as the reference object, assuming that the city has also implemented such a policy in 2015, and the same analytical tools are applied to simulate the assessment. If the results show that the difference between the actual fiscal expenditure data of the city and the fiscal expenditure data estimated through the synthetic control method is relatively small, then it can be inferred that the synthetic control method can effectively evaluate the effect of the community health care combination policy on the fiscal expenditure of Yantai Prefecture-level city. Therefore, two different methods, namely, disposal group replacement and sequential validation, will be used to perform the regional placebo test in this study.

##### 4.2.3.1 Disposal group replacement

First, this study was analyzed using the replacement of the disposal group. Table 1 shows that the synthetic weight of Jinan is 0.923, which means that Jinan and Yantai are highly similar. Therefore, in the disposal group replacement process, Jinan is selected as the disposal group to test the fiscal expenditure status of the synthetic sample and the actual sample before and after the policy implementation, and Yantai Prefecture-level city is removed. Table 4 shows the comparison of Jinan predictor variables with Yantai. Overall, the differences between the Jinan predictor variables and Yantai are small.

**Table 4:** Fitting and comparison of predictor variables.

Variable	Yantai	Jinan
*lngdp (billions)*	8.472	8.275
*lnpopulation (ten thousand people)*	6.543	6.823
*lnfrevenue (ten thousand dollars)*	15.168	14.928
*lnconsumption (dollars)*	10.009	9.587
*lnmedicialexp (ten thousand dollars)*	12.716	12.615
*lnurbancom (ten thousand dollars)*	13.403	12.739

**Figure 5:**
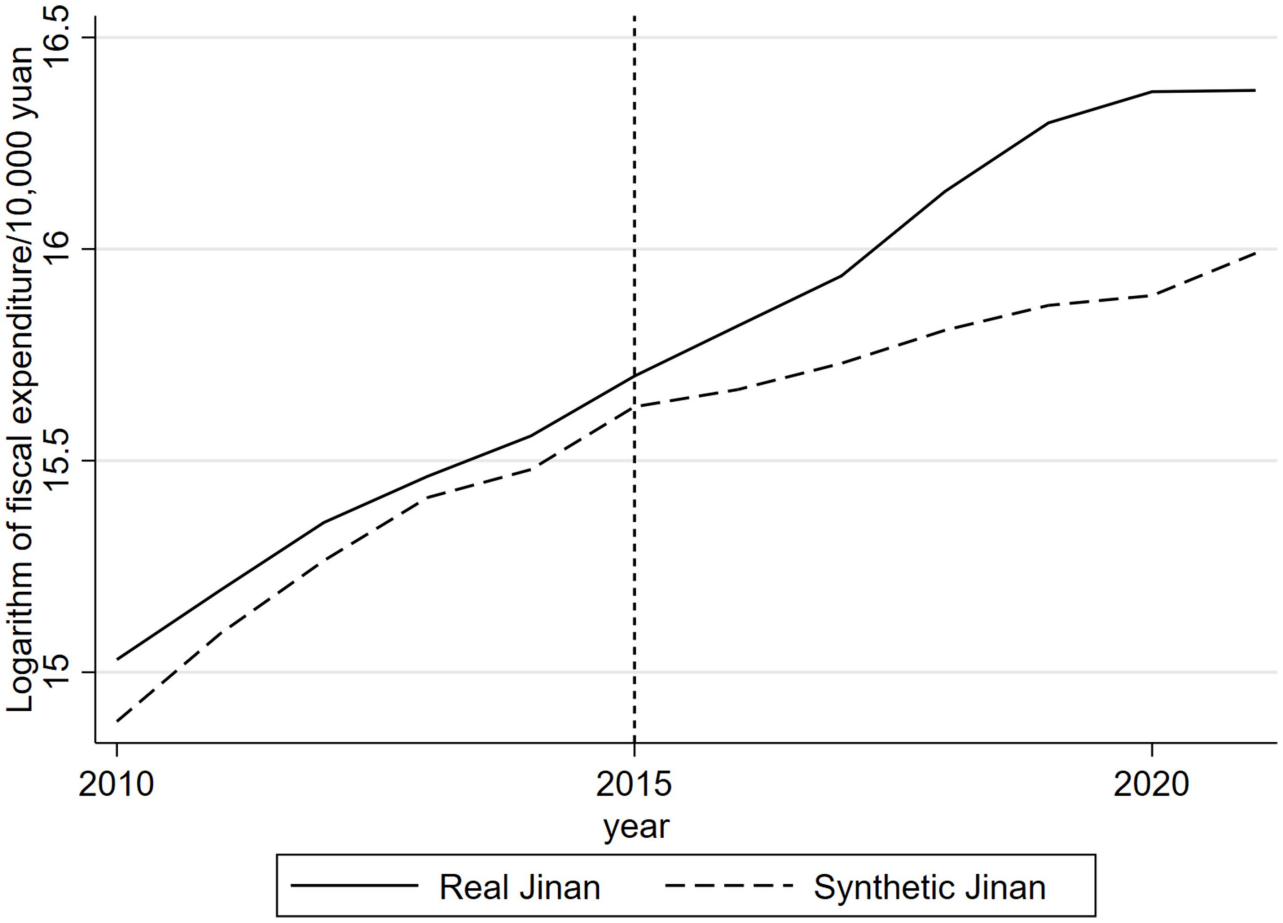
Real and synthetic fiscal expenditure in Jinan.

Figure 5 presents the results of the regional placebo test for Jinan. From the figure, it can be seen that before and after the implementation of the community health care integration policy, Jinan’s real fiscal expenditures as a whole move with the synthetic fiscal expenditures. This indicates that the fluctuation between the real fiscal expenditures and the synthetic sample fiscal expenditures is relatively small, and the trend has not changed significantly and abruptly. To a certain extent, this proves the impact of the policy of combining community health care on fiscal expenditures, while excluding the impact of other contingent factors on the changes in fiscal expenditures.

##### 4.2.3.2 Sort test

Although previous studies have found a reduction in fiscal expenditure after the implementation of the community health care integration policy in Yantai, it was not possible to determine whether the effect of the policy on fiscal expenditure was statistically significant. Therefore, this study was re-validated using the sorting test proposed by Abadie et al. (32). The sorting test aims to explore whether the effect of the implementation of the community health care integration policy is significantly different from zero, which is judged by assessing the probability of consistency between the fiscal expenditures of other cities and the characteristics of Yantai’s fiscal expenditures in the synthetic sample. The basic idea is: based on the 2015 data, assuming that the cities in the control group have also implemented the community health care combination policy, and constructing the simulated fiscal expenditure data of each city with the help of the synthetic control method, so as to estimate the effect of the policy implementation under the hypothetical scenario. Comparing the effect of the actual implementation of the policy in Yantai and the effect of the simulated implementation of the policy in the control group, if there is a significant difference between the two, it means that the policy of combining community healthcare and nursing care has had a significant impact on Yantai’s fiscal expenditures rather than being caused by a random event; and vice versa.

**Figure 6:**
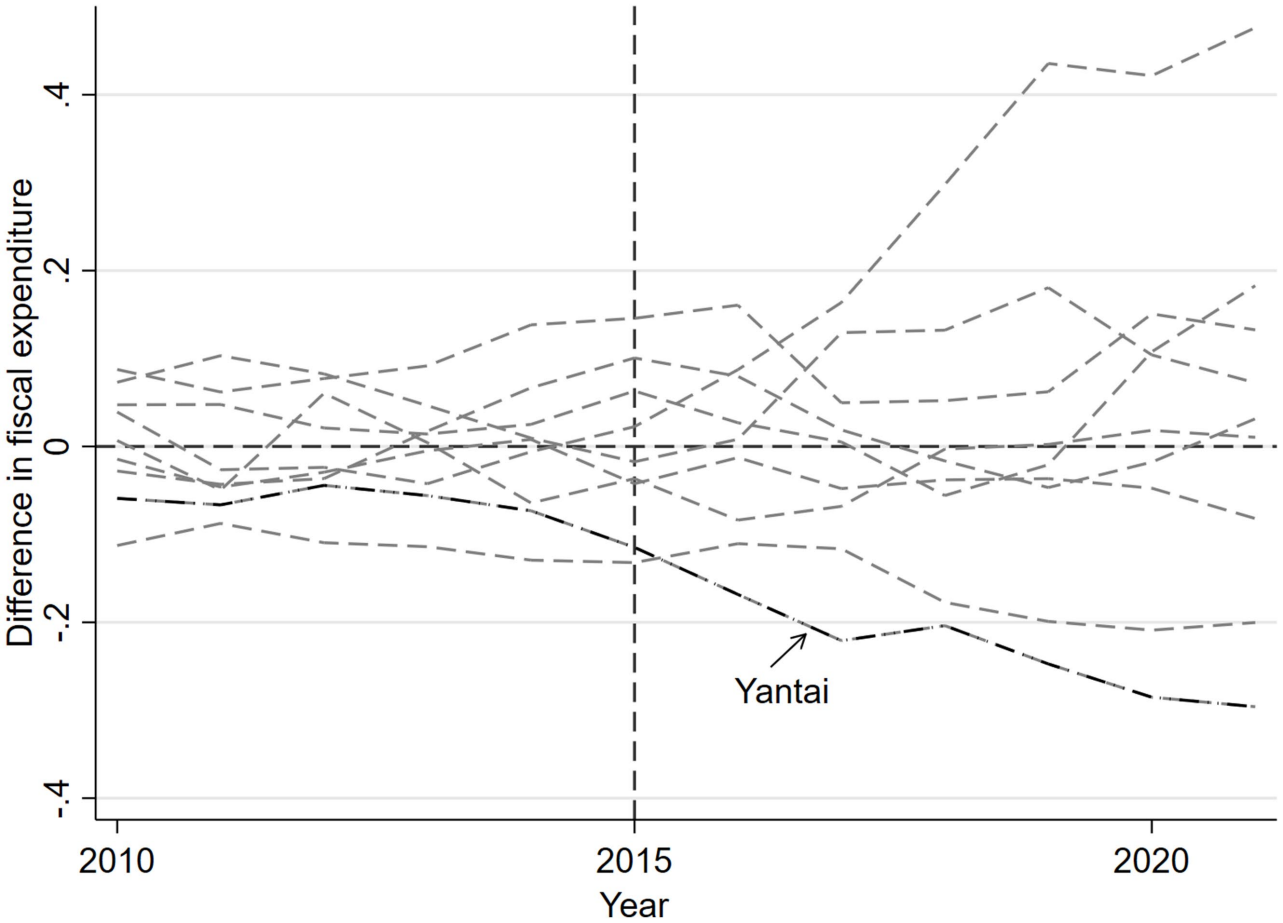
Prediction error distribution in Yantai and other regions.

Drawing on the study of Abadie et al. (32), this study first calculated the mean prediction error (MSPE) for Yantai to be 0.055. In order to improve the estimation accuracy, the control sample was processed: samples with an MSPE value of more than twice the value of Yantai were excluded, for a total of one sample excluded. This sample had poorly fitted the financial expenditure characteristics before the implementation of the community health care integration policy, reducing the accuracy of the explanation of the changes in financial expenditure after the implementation of the policy. In the end, 13 valid samples were retained for the ranking test, and the test results are shown in Figure 6.

From the distribution of the sample MSPE presented in Figure 6, it can be observed that: before 2015, the average prediction error of Yantai’s fiscal expenditures changed by a relatively small amount compared with other regions, and thereafter the gap between Yantai and other regions gradually widened, and the prediction error of its fiscal expenditures was located at the outer boundary of all regions. This suggests that the implementation of the community health care integration policy has a significant impact on Yantai’s fiscal expenditures and that the probability of being significantly different from zero is 7.69%. Therefore, it is reasonable to assume that the reduction in fiscal expenditures in Yantai is significant at the 10% significance level.

### 4.3 Explanation of reasons

Theoretically, the implementation of the policy of integrating community health care and nursing care will help reduce fiscal expenditure and ease the pressure on fiscal payments and the fiscal deficit gap. The policy of integrating community health care and older adult care is a major livelihood project implemented by China in recent years. Under this policy framework, healthcare services and older adult services can be organically integrated, which can not only effectively alleviate the problem of tight medical resources due to aging, but also significantly reduce the government’s fiscal expenditure on healthcare, thus providing a feasible way to reduce the overall fiscal burden and narrow the fiscal deficit.

Firstly, from the perspective of improving the efficiency of healthcare services, the strategy of “integrating healthcare in the community” emphasizes the importance of the role of primary healthcare institutions and their capacity building. Traditionally, many older adult people tend to go to large hospitals when they are sick, which undoubtedly increases the pressure on medical institutions in urban centers and also leads to a certain degree of waste of resources - as not all diseases require a high level of specialized treatment. By strengthening the functional positioning of community health service centers and equipping them with the necessary medical equipment and professional staff training, it has become possible to make it possible for common illnesses to be properly treated at home. This not only reduces the inconvenience caused by patients running back and forth, but also greatly improves the level of primary medical services; more importantly, it prompts the spread of quality medical resources originally concentrated in a few large general hospitals to a wider area, realizing a more equitable and reasonable distribution pattern (38). In addition, with the development and application of information technology, new types of services, such as remote consultation, have been introduced into the daily diagnostic and treatment activities, further enhancing the problem-solving ability at the community level.

Secondly, in response to the unique health management needs of the older adult, the “Community Healthcare Integration” program pays special attention to building a comprehensive service system covering preventive healthcare, chronic disease management and rehabilitation care (39). Specifically, it encourages and supports the establishment of close cooperation between various types of older adult care institutions and local hospitals, and jointly carries out regular medical checkups, health lectures and other activities, so as to help the older adult keep abreast of changes in their own conditions; and for patients who have been diagnosed with chronic diseases or are in the recovery period, it provides personalized tracking and follow-up plans as well as the necessary technical support (e.g., medication delivery to the home) (40). This seamless care model not only ensures that each older adult person receives the care that best suits his or her condition, but also prevents the deterioration of their condition that would result in hospitalization to a large extent, which improves the quality of life of the older adult and reduces the burden of medical care and medical expenses, than reduces the proportion of government expenditure on healthcare.

Lastly, “community healthcare integration” is also committed to promoting the development of the entire industry in the direction of greater transparency and standardization. On the one hand, government departments have guided the market order toward a virtuous cycle by combating various forms of fraud and formulating a scientific and reasonable standard price catalog, which is conducive to curbing the phenomenon of excessive medical care and promoting the rationalization of medical service prices. On the other hand, the active introduction of preferential policies to attract more social capital into the field, such as measures to reduce or waive some taxes and fees and to prioritize the use of land, has greatly stimulated the endogenous participation of all sectors of society. With the gradual implementation of these reform measures, Chinese urban and rural residents, especially the older adult, will enjoy high-value medical services.

## 5 Research conclusion and countermeasure suggestion

In recent years, community healthcare integration policies have been widely implemented in various regions, followed by a slew of new community healthcare integration models, including healthcare integration and community-based services. The implementation of community healthcare integration policy influences numerous elements and patterns of fiscal expenditures; therefore, understanding how it affects fiscal expenditures is an important theoretical and empirical subject. This study uses the adoption of a community healthcare integration policy in Yantai Prefecture-level city, China, in 2015 as a natural experiment and uses the synthetic control method to examine the influence of the policy on fiscal expenditures. Meanwhile, the estimation results are successfully validated using double difference models, time placebo, and area placebo tests. Finally, alternative explanations for the cost-saving effect of the community healthcare integration program on fiscal expenditures are investigated. The community healthcare integration policy reduces fiscal expenditures by lowering government healthcare spending in the pilot cities, and the robustness test confirms that the implementation of the community healthcare integration policy is the cause of the fiscal expenditure reduction in the pilot cities, ruling out the influence of other chance factors.

This research furnishes crucial insights regarding fiscal sustainability in aging societies. On one hand, our study verifies the “fiscal pressure - relieving” effect of the policy. The case of Yantai reveals that, after the implementation of the policy, the average annual fiscal expenditure has decreased by 6.6%. This implies that if the policy is extended nationwide, it could liberate a fiscal space amounting to hundreds of billions. We propose incorporating the integration of medical and older adult care into the transfer - payment assessment system. Regions with remarkable policy - implementation results should be given special incentives, thus forming a virtuous cycle of “fiscal savings - livelihood improvement.” On the other hand, the research discovers that there exists a lag in the policy effect. This indicates that local governments should establish a multi - year fiscal - budgeting mechanism. It is recommended to set up a special transitional fund in the initial stage of policy implementation. This fund can ease the short - term expenditure pressure on grassroots medical institutions for equipment procurement and professional training, ensuring the sustainability of the policy effect.

Based on this, local governments at all levels in the new round of promotion of community healthcare integration should adopt appropriate implementation strategies according to the actual situation in order to realize the benign and sustainable development of community healthcare integration and reduce the fiscal burden. Specifically, it can start from the following aspects:

First, optimize resource allocation and improve service capacity. To ensure the effective functioning of community healthcare systems, local governments must enhance support for primary healthcare institutions. This includes increasing financial investment, improving infrastructure, introducing advanced equipment, and strengthening the professional training of healthcare personnel, among other measures. Special funds may be established to facilitate the construction and operation of community health service centers, as well as to promote the creation of comprehensive health management platforms in regions equipped to offer one-stop services. Simultaneously, it is essential to focus on developing a high-quality medical and nursing team by consistently enhancing their professional skills and service levels through regular training courses and academic exchanges. Additionally, exploring the establishment of a cross-regional cooperation mechanism can facilitate the allocation of high-quality medical resources to less-developed areas, thereby reducing urban–rural and regional disparities.

Second, improving the policy and regulatory system. Perfect laws and regulations are important prerequisites to ensure the smooth promotion of community health care integration. Therefore, the national level should speed up the formulation and improvement of relevant laws and regulations to provide a solid institutional guarantee for the development of this field. Clarify the division of responsibilities between governments at all levels and relevant institutions, and refine the operation process of each policy measure; establish and improve the supervision and evaluation mechanism to track and manage the implementation of the project, so as to identify and solve problems in a timely manner; include the level of community healthcare and nursing care services in the assessment standard of special transfer payments for senior care services, and build a “positive incentive-negative constraint” regulatory mechanism; and strengthen the specification and guidance of the market to prevent unhealthy competition, and to maintain a good industry order. In addition, taking into account the different levels of economic and social development in different regions, it is recommended that local characteristics and practical needs be fully considered in the legislative process, and that local governments be given a certain degree of autonomy so that they can flexibly adjust specific practices on the basis of following basic principles.

Third, to promote the construction of information technology. The application of informatization is of great significance in upgrading the level of community medical services. On the one hand, a unified information platform can be constructed to realize data sharing and exchange, break down departmental barriers and improve work efficiency. On the other hand, the use of big data analysis technology can help managers better understand the health status of the older adult and the trend of service demand, so as to make more scientific and reasonable decisions. As the concept of “Internet Plus” medical and health care gradually takes root in people’s hearts, new types of services such as remote diagnosis and treatment and online consultation are becoming increasingly popular. Therefore, relevant departments should actively promote such applications and incorporate them into the regular medical service system. However, it should be noted that in the process of promoting informatization, attention needs to be paid to the issue of personal information security, and effective means should be adopted to protect patients’ privacy from being leaked.

Fourth, the active introduction of social forces. In addition to relying on the government to lead, but also needs to fully mobilize the enthusiasm of all sectors of society to participate in the community health care integration business. Therefore, the government can attract more social capital to enter the field of investment and business through the introduction of preferential policies, such as reducing or waiving part of the tax, providing low-interest loans or gratuitous subsidies to reduce the pressure on business costs. At the same time, it is also necessary to strengthen support for social organizations and individual volunteers, encouraging them to carry out various forms of public welfare activities, so as to form a good atmosphere of concern and care for the older adult in society as a whole. In addition, diversified financing channels can be explored, such as the issuance of special bonds and the establishment of funds to raise funds for supporting project construction and development.

This study has achieved some results in exploring the impact of community health care integration policies on fiscal expenditures. Nevertheless, data constraints hinder the assessment of the long-term effects of community healthcare integration programs on financial expenditures. Specifically, this study looked for panel data for 14 cities including Yantai City from 2010–2021, but there are only seven observation years (2015–2021) after the implementation of the community healthcare and nursing combination policy, the implementation of the community healthcare and nursing combination policy is still short, and in the stage from nothing to something, the number of people who participate has gradually increased, releasing the demand for healthcare, and the effectiveness of healthcare may take longer to present itself, so only the short-term financial impact of the community healthcare integration policy can be assessed. In order to understand the long-term effects of the community healthcare integration policy more comprehensively, future research can consider the following two aspects: firstly, extending the observation period and collecting data from more years in order to analyze the long-term trends and impacts of the policy implementation. Conversely, the amalgamation of case studies, interviews, and other qualitative research, along with a comprehensive analysis of the implementation outcomes and insights derived from the community healthcare integration policy across various regions and socio-economic contexts, can furnish policymakers with a more nuanced foundation for decision-making. Through these comprehensive research methods, it is conducive to a more comprehensive assessment of the long-term effects of the policy of combining community health care and nursing care, and provides scientific guidance and recommendations for the sustainable and healthy development of the older adult care business.

## Data Availability

Publicly available datasets were analyzed in this study. This data can be found at: https://data.cnki.net/yearBook/single?id=N2024030190&pinyinCode=YSDTJ.
